# Detection of asymptomatic SARS-CoV-2 infections in daycare centers, schools, and companies for regional pandemic containment by a PCR testing laboratory cooperative between July 2021 and June 2022

**DOI:** 10.3205/dgkh000425

**Published:** 2022-12-06

**Authors:** Ralph Bertram, Laura Grebenstein, Stefanie Gualdi, Bernd Seibold, Ralf Birkmann, Klaus Korn, Johannes Bisping, Ralf Schabik

**Affiliations:** 1 Klinikum Nürnberg, Universitätsinstitut für Klinikhygiene, Medizinische Mikrobiologie und Klinische Infektiologie, Paracelsus Medizinische Privatuniversität, Nürnberg, Germany; 2 Wirtschaftskraft Nürnberger Land GmbH, Lauf a.d. Pegnitz, Germany; 3 Bisping & Bisping GmbH & Co. KG, Lauf a.d. Pegnitz, Germany; 4 Universitätsklinikum Erlangen, Virologisches Institut, Friedrich-Alexander-Universität Erlangen-Nürnberg, Erlangen, Gremany; 5 A.S.S. Apotheker. Service. Schabik., Altdorf bei Nürnberg, Germany

**Keywords:** SARS-CoV-2, COVID-19, coronavirus, pool testing, PCR test laboratory

## Abstract

As an important element in the regional containment of the COVID-19 pandemic a PCR testing laboratory with a cooperative character was founded in spring 2021 to screen for SARS-CoV-2 in the Nuremberg region, Germany. The aim was to detect asymptomatic infections in day care facilities for children, schools, and companies. The laboratory used an established RT-PCR protocol and analyzed approximately 18,500 pools of up to 25 pooled samples each from gargles or swabs (“lollipops”) from up to 135 facilities between July 2021 and June 2022. Usually, the participating facilities were informed about positive pools within a few hours. Retention samples from positive pools were usually analyzed on the same day, and the results were reported to the facilities as well as the German Electronic Reporting and Information System (DEMIS). In the laboratory results, both the local incidences and the transition from the Delta- to the Omicron surge in early 2022 were well reflected. It is plausible that about 4,800 secondary infections could be prevented from the approximately 1,570 positive individual samples detected in conjunction with appropriate isolation measures. Such a PCR laboratory, which is characterized by short response times and high flexibility, can thus provide valuable services for regional surveillance of infection incidence.

## Abstract (de)

Als ein wichtiger Baustein zur regionalen Eindämmung der COVID-19 Pandemie wurde im Frühjahr 2021 ein PCR-Testlabor mit genossenschaftlichem Charakter zum Screening auf SARS-CoV-2 in der Region Nürnberger Land gegründet. Ziel war die Detektion asymptomatischer Infektionen in Kindertagesstätten, Schulen und Unternehmen. Das Labor nutzte ein etabliertes RT-PCR Verfahren und analysierte zwischen Juli 2021 und Juni 2022 aus bis zu 135 Einrichtungen rund 18.500 Pools mit je bis zu 25 vereinigten Proben aus Gurgelaten bzw. Abstrichtupfern („Lollis“). In der Regel wurden die teilnehmenden Einrichtungen innerhalb weniger Stunden über positive Pools informiert, Rückstellproben positiver Pools wurden meist noch am selben Tag analysiert und die Ergebnisse an die Einrichtungen sowie das Deutsche Elektronische Melde- und Informationssystem (DEMIS) gemeldet. In den Laborergebnissen bildeten sich über den Zeitraum Juli 2021 bis Juni 2022 sowohl die lokalen Inzidenzen als auch der Übergang von der Delta- zur Omikron-Welle Anfang 2022 gut ab. Es ist plausibel, dass sich bei den ca. 1.570 detektierten positiven Einzelproben in Verbindung mit geeigneten Isolationsmaßnahmen etwa 4.800 Sekundärinfektionen verhindern ließen. Ein solches PCR-Labor, das sich durch kurze Antwortzeiten und hohe Flexibilität auszeichnet, kann somit wertvolle Dienste für die regionale Überwachung des Infektionsgeschehens leisten.

## Introduction

The Severe Acute Respiratory Syndrome Coronavirus 2 (SARS-CoV-2) [1], first detected in January 2020, is the causative agent of Corona Virus Disease 2019 (COVID-19), which was classified as a pandemic on 11 March 2020 [2]. Already on 27 February 2020, the German Federal Ministry of Health declared that the epidemic had reached Germany [3]. By early November 2022, over 35 million COVID-19 cases and around 153,000 deaths had been reported to the Robert Koch Institute in Germany [4]. At the beginning of the pandemic, drastic measures were adopted in Germany to contain the incidence of infection, such as curfew restrictions and the closure of schools and day care facilities for children. It was known from earlier studies that the risk of transmission of viral diseases in day care centers is favored by being in close groups and that the occurrence of various infectious diseases in the population is often associated with infectious events in day care centers [5], [6]. However, school and day care closures can seriously affect the physical and mental health of children and adolescents [7]. In order to counteract repeated large-scale school closures in the SARS-CoV-2 pandemic, various surveillance programs were also initiated in Germany as pilot projects or implemented on a larger scale. The STACAMA and STACADO studies aimed at preventing outbreaks at schools in Magdeburg and a boarding school in Regensburg, respectively [8], [9]. The WICOVIR study involved schools in Bavaria [10], and the B-FAST study schools in North Rhine-Westphalia [11], [12], [13]. These studies were based on nucleic acid-based methods for the detection of SARS-CoV-2 infections, which are significantly more sensitive and specific than antigen tests and can thus detect viral genetic material even before an infected carrier becomes infectious [14]. Compared to antigen tests, however, PCR tests are more complex and require more equipment, which is why they are mainly carried out by academic institutes and commercial laboratory service providers. Sampling can be done by swabbing, throat rinsing (gargling) or collection of saliva (e.g. by “lollipop tests”) [9], [12]. This article describes the establishment of a PCR laboratory for regional pandemic containment, and the laboratory's experience in the SARS-CoV-2 pandemic between July 2021 and June 2022. 

## Motivation and initiative for founding the test lab

In the spring of 2021, members of the “Führungsgruppe Katastrophenschutz Nürnberger Land” (involved in disaster control in the district Nürnberger Land) developed a testing concept to accompany the pandemic situation, in which PCR tests also played a central role. In anticipation of an overload of existing commercial laboratories, three private individuals and seven mid-sized companies founded the “Wirtschaftskraft Nürnberger Land GmbH” in May 2021 to establish and operate a PCR laboratory as quickly as possible. The management, working on a voluntary basis, did not pursue profits. The district administrator (“Landrat”) of the district Nürnberger Land, the parties represented in the district council, as well as several authorities welcomed and supported the idea. The initiators were united by the mission to monitor the regional SARS-CoV-2 infection incidence through closely-timed PCR screening of asymptomatic persons from public and private institutions on a fact-based basis and to contain it if possible. One important rationale behind this was to prevent school, kindergarten and daycare-center closures. Companies were also given the opportunity to participate in the screening tests. The PCR laboratory was set up in the premises of a health facility in the district of Nürnberger Land and went into operation in July 2021 using the WICOVIR IT infrastructure [10]. With the start of the school year 2021/2022, the intent was to test up to 35,000 children twice weekly. 

## Technical and legal specifications

After completion, the laboratory had, among other things, two safety cabinets, a UV cabinet for PCR-specific work and further equipment for a molecular biology detection laboratory. Consumables and molecular biological reagents were purchased from established manufacturers and distributors. The laboratory was designed as an environmental laboratory for low-threshold PCR testing for SARS-CoV-2, approved for biological safety level 2 and certified by the German testing company Dekra according to DIN EN ISO 9001. The sample material sent in by the participating facilities consisted of pooled gargles (“gargle pools”) or swabs (“lollipop pools”) with a maximum of 25 samples per pool. In case of a positive signal in a pool, all retention samples of the individuals represented in the pool were retested. The samples from the participating institutions were usually delivered by courier to the laboratory within 90 min, sometimes in less than 30 min, after sample collection. SARS-CoV-2 RNA was detected by an established, freely available quantitative real-time PCR (RT-PCR) protocol [15]. The laboratory processed the samples with a low level of automation, from collection and processing to preparation of the reaction mixtures and evaluation of the RT-PCR signals. The centerpiece was two qTOWER³ RT-PCR thermal cyclers (Analytik Jena, Germany) for reactions in 96-well format. The capacity of the laboratory was about 700 pooled or individual samples per day. The sending institutions were informed of the results of the pool tests immediately after completion, usually in the afternoon on the day of the test, so that isolation measures for groups could already be taken if necessary. After pool resolution, the positive individual results were usually also communicated to the senders on the day of the test and additionally reported to the German Electronic Reporting and Information System (DEMIS) before 7 a.m. of the following day, so that health authorities and the Robert Koch Institute were informed quickly and digitally about positive detections. For some of the positive samples, variant testing was carried out at the Institute of Virology of the University Hospital Erlangen. To this end, the SARS-CoV-2 variant I, variant IV and variant VII assays from Seegene (Seoul, Republic of Korea) were used. 

## Participating institutions and development of the pool number

**Figure 1:**
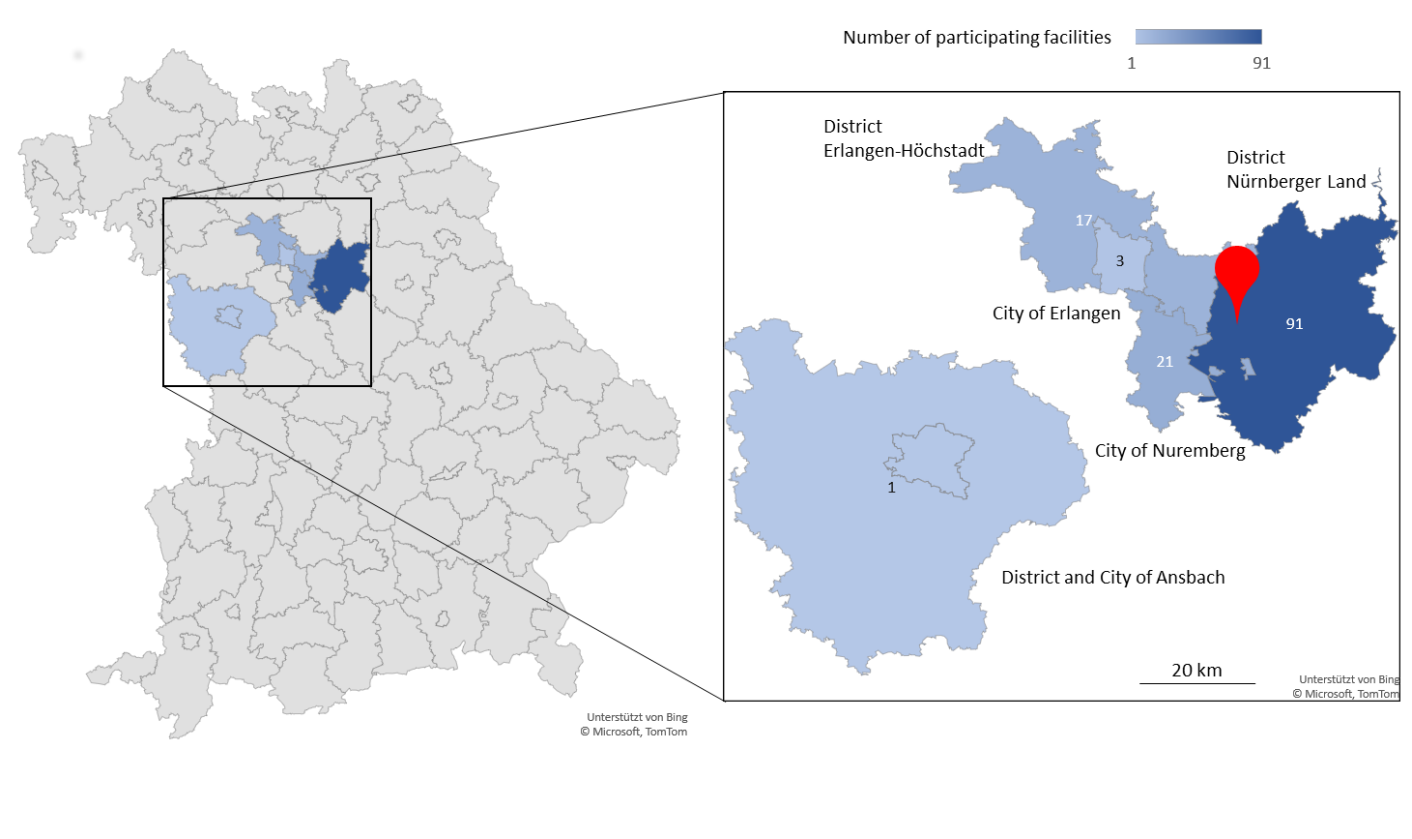
Catchment area of the participating facilities in Northern Bavaria. Detail: Colour-coded representation of the cities and districts according to the number of participating facilities. Red marker: location of the PCR laboratory

**Figure 2:**
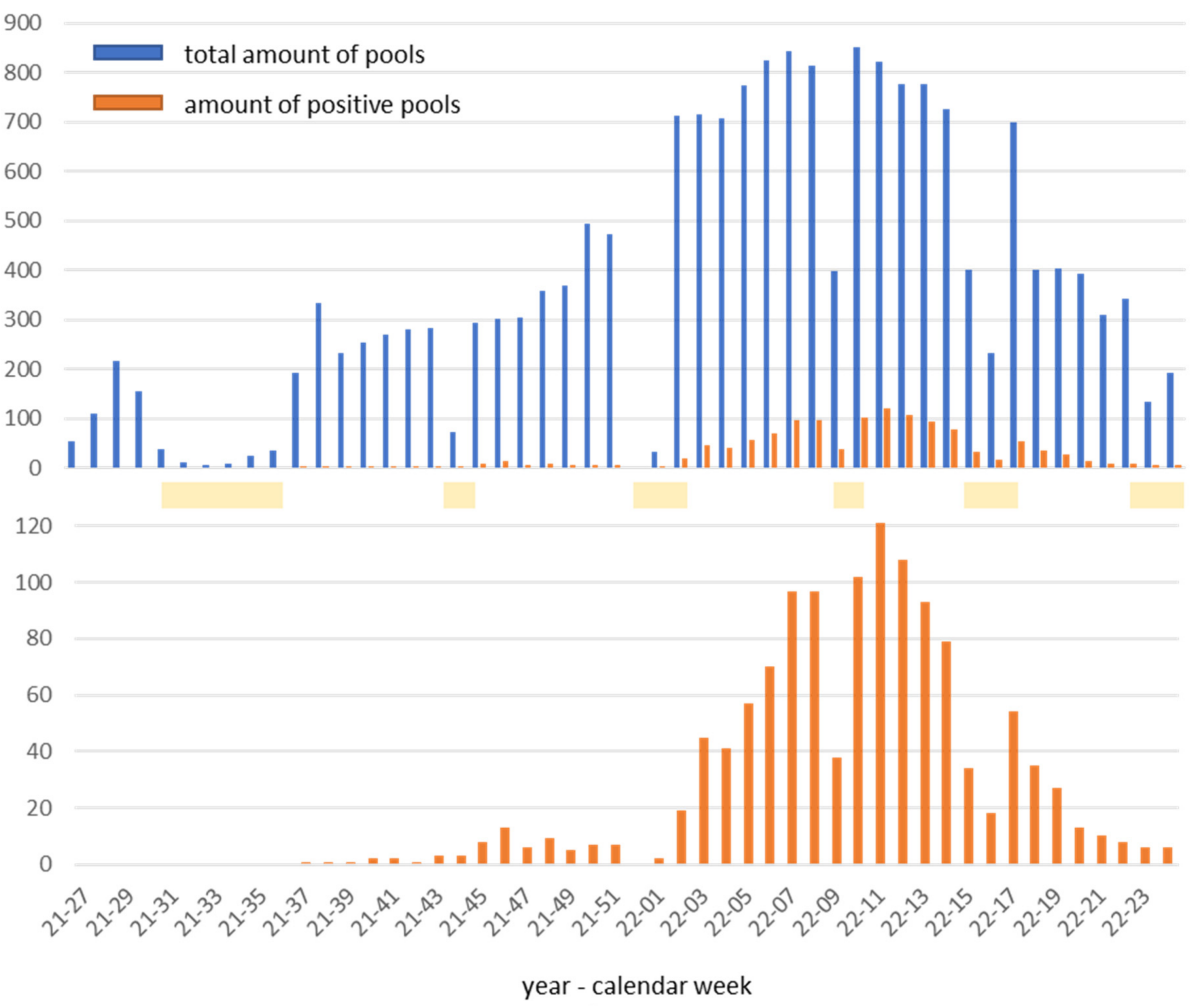
Total number of pools and number of positive pools (below: enlarged scale). Holiday periods highlighted in yellow

Around 135 facilities in the districts Nürnberger Land, Erlangen-Höchstadt, and Ansbach, as well as the cities of Nuremberg and Erlangen took advantage of the PCR laboratory's screening offer. This means that the catchment area for the laboratory was about 3,600 km^2^ (Figure 1 (Fig. 1)). Most of the facilities were kindergartens and daycare centers of various institutions as well as schools and private companies. The latter bore the costs for the screening themselves, while in the other cases, the district Nürnberger Land or the state of Bavaria took over the financing. Two tests per week and institution became established as an adequate frequency, as also confirmed by the literature [11], [12]. The number of pools tested per week increased from 54 to 218 in the first weeks of the laboratory’s operation from early to mid-July 2021. During holiday periods, pool numbers mostly decreased significantly and increased to peaks around 850 per week by February/March 2022 (Figure 2 (Fig. 2)). With the phasing out of the no-cause testing regulations for students, the number of pools tested dropped from mid-March 2022. By mid-June 2022, the number of pools tested was just under 200 per week. In total, the laboratory tested around 18,500 pools for SARS-CoV-2 RNA between July 2021 and mid-June 2022. Around 1,250 pools and, from these, around 1,570 individual samples were RT-PCR positive. 

## Positive rates and SARS-CoV-2 variants in the pandemic course

**Figure 3:**
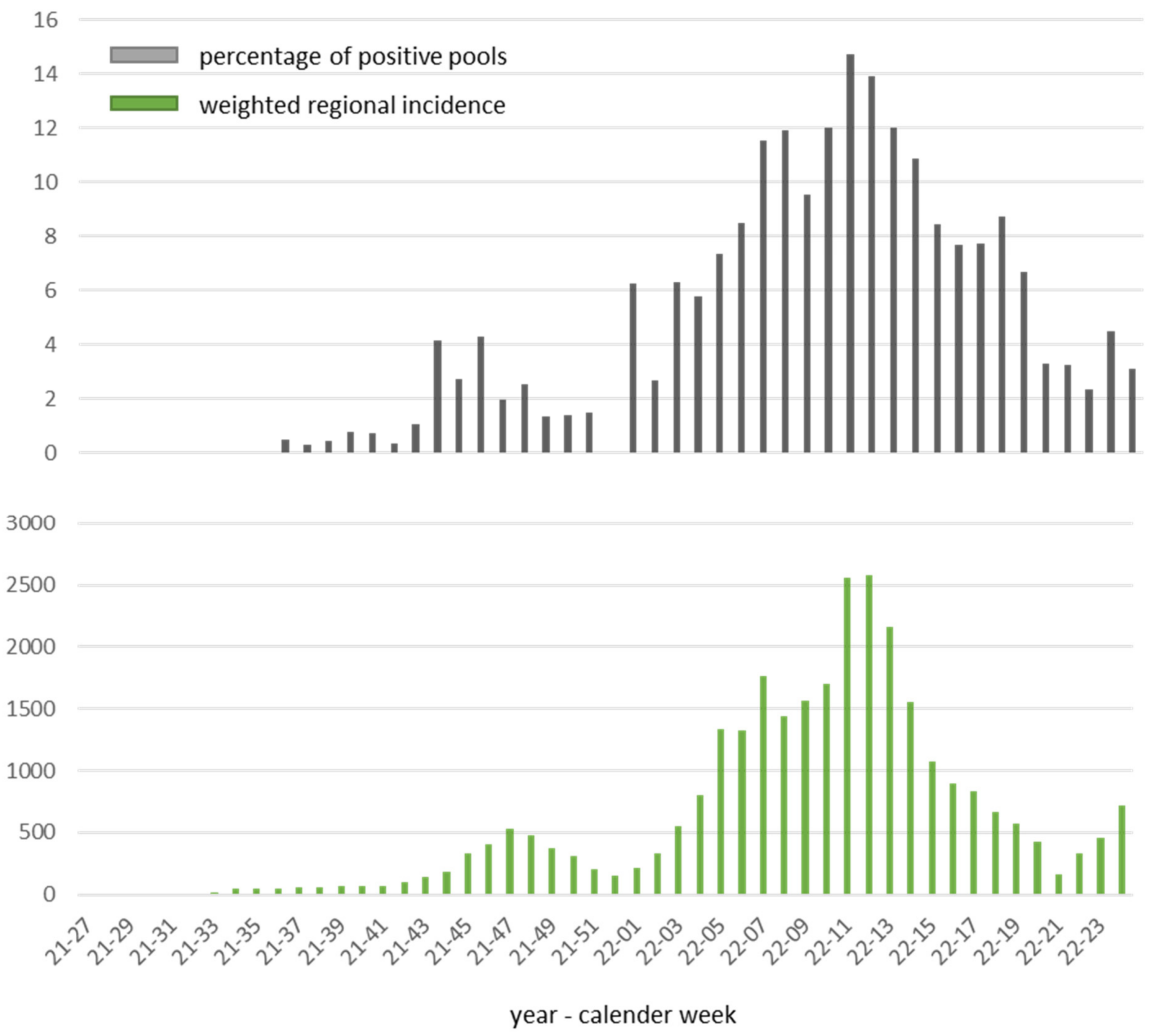
Top: positive rate of the pools, bottom: weighted incidences of the districts and cities according to the number of participating facilities (except for the city of Erlangen and the district of Ansbach due to insufficient numbers)

**Figure 4:**
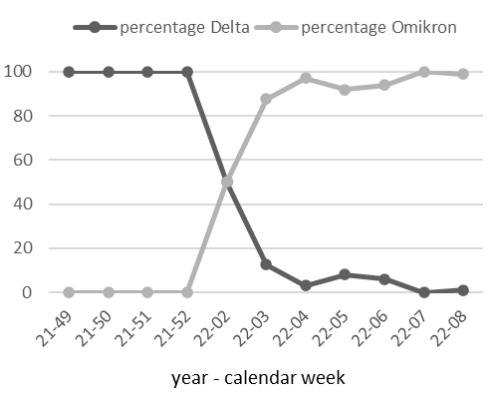
Displacement of the Delta by the Omicron variant based on the laboratory results

Between the beginning of July and the beginning of September 2021, all 665 pools tested were negative. By mid-October 2021, positive rates were below 1%. The highest rates of positive pools were recorded between mid-January and the end of May 2022, with a peak of 14.7% in calendar week 11 (14–20 March 2022). The comparison with the regional incidence values in the comparison periods [16] shows a very good agreement of the courses (Figure 3 (Fig. 3)). Random testing of positive samples for SARS-CoV-2 variants using mutation-specific PCR assays impressively revealed the displacement of the Delta variant by the Omicron variant at the turn of the year 2021/2022 [17] (Figure 4 (Fig. 4)). While between the end of November and the end of December 2021 all variant-tested samples were assigned to the Delta variant (21/21), the share of the Omicron variant was already 50% (1/2) in the second calendar week of 2022, 87.5% (14/16) in week 3/2022 and moved to over 90% in the following five weeks. After that, variant testing was discontinued for cost reasons due to the dominance of the Omicron variant. 

## Benefit assessment of the testing laboratory regarding regional infection protection

Of course, a screening regime like that of this testing laboratory does not protect against primary infections. However, early isolation of infected persons can prevent secondary infections [18]. In the following, we will roughly quantify how screening may have contributed to the prevention of secondary infections in the region. According to the studies, the secondary infection rates are around 18% for the Delta variant and around 27% for the Omicron variant [19]. Accordingly, a person infected with Delta infects an average of 0.54 other persons within a four-person household (with Omicron: 0.81), and a person infected with Omicron infects an average of 5.1 other persons (with Delta: 3.4) within a 20-person group with close contact (such as a child in a kindergarten). Especially the latter dynamic can be efficiently prevented by early isolation of infected children from the group. The groups of people sampled by the laboratory are predominantly made up of students, kindergarten and daycare children and, to a lesser extent, employees. As a rough overall estimate, an average factor of ca. 3 for secondary infections per infected person should be plausible here (details of the calculations upon request). With a total of just under 1,600 individual cases of SARS-CoV-2 detected by the laboratory and optimal prevention of secondary infections, this means in this model calculation there were approx. 4,800 potentially prevented infections in the period July 2021 to mid-June 2022. 

## Conclusions

Thanks to the great commitment and cooperation of relevant local individuals and companies, it was possible within a few weeks to recruit specialized staff, equip a PCR laboratory for SARS-CoV-2 detection that was suitable for the regional needs, and establish standardized work processes that meet international quality management criteria. Among other things, the laboratory was characterized by good accessibility in case of queries, high flexibility, proximity to the facilities, and the authority to report to DEMIS. Due to short transport routes and rapid sample processing, facilities with a morning sampling usually received reports of positive pools by lunchtime; positive individual samples were usually identified and reported the same day. This meant that, if necessary, initial measures could be taken just a few hours after sampling. For a suitable balance between transmission prevention and logistic effort, sampling on two days per week was chosen, in line with similar studies that investigated weekly testing frequency [11], [12]. Limitations of the laboratory include the limited scalability (spatial and capacity limitations, little automation). Also, due to the lean workflow in the PCR laboratory, no in-house variant testing of positive samples was possible. As with other laboratory service providers, the imponderables include pre-analytics, i.e. the kind of sampling. Thus, in line with literature [13], the results seemed to be more reliable if the samples were not taken at home but under supervision in the respective facilities. Having a PCR laboratory with a cooperative character available on site offers the possibility to react promptly and effectively to future corona waves or further infectious diseases with epidemic or pandemic potential. This can make a significant contribution to the containment of regional infectious events.

## Notes

### Competing interests

The authors declare that they have no competing interests.

### Ethics 

The Ethics Committee of the Bavarian Medical Association has confirmed that there was no obligation to consult for this report according to § 15 of the Professional Code of Conduct of Bavarian Physicians.

### Acknowledgement 

We would like to thank all employees and trainees of the PCR laboratory and all associates of Wirtschaftskraft Nürnberger Land GmbH. We would like to thank Dr. Ulrike Artmeier-Brandt (Ethics Committee of the Bavarian Medical Association) for the ethical evaluation of the study. Finally, we thank Prof. Dr. Jörg Steinmann for his critical review of the manuscript.

### Authorship

Ralph Bertram and Laura Grebenstein are to be regarded as equal first authors.

### ORCID

Ralph Bertram’s ORCID ID is: 0000-0003-0654-6381

Klaus Korn’s ORCID ID is: 0000-0003-1891-2107
